# Screening eating disorders among workers from an electricity company

**DOI:** 10.1192/j.eurpsy.2025.1411

**Published:** 2025-08-26

**Authors:** I. Sellami, A. Abbes, A. Hrairi, M. A. Ghrab, A. Feki, A. Meska, M. Tah, M. Hajjaji, M. L. Masmoudi, K. Jmal Hammami

**Affiliations:** 1 Occupationnal medecine; 2 Rheumatology, CHU Hedi Chaker, Sfax, Tunisia

## Abstract

**Introduction:**

Eating disorders (EDs) can significantly worsen one’s physical and psychological well-being. In the professional environment, EDs may have serious consequences on the psychological and physical health of workers.

**Objectives:**

Our study aims to screen EDs and psychological health among workers.

**Methods:**

We conducted a descriptive, analytical, cross-sectional survey among workers from an electricity company. We collected sociodemographic and professional characteristics. We screened EDs using the SCOFF questionnaire (Sick, Control, One stone, Fat, Food). Psychological health of our participants was assessed using the Hospital anxiety and depression scale (HADS).

**Results:**

Our study included 497 workers with an average age of 42.2 ± 11.1 years. Among them, 381 were male (76.7%) and 72.2% were married. Two hundred twenty-two participants engaged in physical activity, and 44.1% were smokers. The average job tenure was 15.2 ± 10.8 years. According to the SCOFF questionnaire, 35.6% of participants showed signs of possible eating disorders (EDs). Additionally, 12.5% of participants were found to be anxious, and 13.9% were depressed. The likelihood of EDs was higher among those with depression. Factors associated with an increased probability of EDs included feeling slowed down, experiencing anxiety symptoms like ‘butterflies’ in the stomach, and losing interest in personal appearance.

**Conclusions:**

Eating disorders are common among workers, with notable rates of anxiety and depression also observed. It is essential to raise awareness about the importance of a healthy, balanced diet to support mental well-being and, in turn, improve workplace productivity.

**Disclosure of Interest:**

None Declared

